# The impact of digital transformation of infrastructure on carbon emissions: Based on a "local-neighborhood" perspective

**DOI:** 10.1371/journal.pone.0307399

**Published:** 2024-07-18

**Authors:** Jinzhao Song, Qiyue Gao, Xiangxiang Hu, Jie Lei

**Affiliations:** School of Management, Xi’an University of Architecture and Technology, Xi’an, China; Zhejiang Sci-Tech University, CHINA

## Abstract

In light of the recent worldwide scientific and technological revolution, it is imperative that urban infrastructure undergo a digital transformation in order to lower carbon emissions and support sustainable urban growth. However, to date, there is a lack of empirical research on carbon emissions based on the digital transformation of urban infrastructure. This paper uses data from 178 prefecture-level cities in China from 2005 to 2020 to study the impact of digital transformation of urban infrastructure on carbon emissions based on the "local-neighbourhood" perspective using a spatial difference-in-differences model. The results show that the digital transformation of urban infrastructure reduces the intensity of local carbon emissions while also reducing the carbon emissions of neighbouring cities, with a spatial spillover effect, and the boundary of this spatial spillover is 600 km. Mechanistic analyses suggest that digital transformation of urban infrastructure can reduce carbon emissions locally as well as in nearby areas by promoting green technological innovations. In light of this, this study has important policy implications for maximising the contribution of digital transformation of infrastructure to reducing carbon emissions.

## Introduction

Environmental, social, and economic systems are already significantly impacted by the problem of global warming brought on by carbon emissions [1]. China has a duty and responsibility to foster low-carbon economic development and mitigate global climate change, given its status as the world’s major carbon emitter [2]. Consequently, the 2030 carbon peak and 2060 carbon neutrality strategic goal has been put forth by the Chinese government [3]. As the major vehicle of human production and life, cities are not only the primary drivers of regional economic growth, bringing about population and industrial agglomeration, but also a major source of carbon emissions. In light of this, how to lower carbon emissions at the municipal level has emerged as a crucial tool for meeting the "dual-carbon" goal. As China’s economy enters a high-quality growth stage, the traditional infrastructure approach to economic development with high energy consumption and pollution can no longer reduce carbon emissions or provide sufficient help for China to achieve its "dual-carbon" goal. The rapid growth of new digital technologies represented by artificial intelligence, 5G, the Internet of Things and the industrial Internet in the context of the development of a new wave of global scientific and technological revolution, achieving the deeper integration and application of innovations in the fields of resources, energy, environment and other areas [4]. Digital infrastructure, centred on a new generation of information technology, has emerged to play a more critical role in combating climate change. According to China’s Government Work Report in 2021, industrial digital transformation and the industrialization of digital industries must be jointly promoted in order to accelerate digitization’s growth, develop new benefits for the digital economy, and create synergies. Furthermore, China has also suggested to "promote the new infrastructure construction and traditional infrastructure in a coordinated manner", and to facilitate the digital transformation of traditional infrastructure to achieve sustainable urban development [5]. It is evident that digital transformation has gradually turned into a new driving force for the conversion of old and new kinetic energy, the transformation and upgrading of traditional industries, and the realization of high-quality development in China’s economy [6]. In this context, it is of theoretical and practical significance to explore the relationship between infrastructure digital transformation on carbon emissions. Digital transformation is an all-encompassing, multi-faceted, whole-chain transformation process utilizing digital technology for the vast majority of traditional industries [7]. As the foundation for digital transformation, digital technology acts on cities’ traditional infrastructure to create digital infrastructure and digital infrastructure transformation, which in turn fosters the urban digital economy and impacts the urban environment [8], directly or indirectly lowering urban carbon emissions [9]. Therefore, digital transformation has become a powerful way to realize the goal of "dual carbon".

While data and policies have validated that digital transformation of infrastructure can reduce urban carbon emissions, can digital transformation of urban infrastructure have spatial spillover effects on urban carbon emissions? If so, what is the mechanism of its impact? The main purpose of this study is to try to answer the above questions through a combination of theory and empirical evidence, and to provide policy recommendations for achieving the "dual-carbon" goal and promoting sustainable urban development. Therefore, this study constructs a theoretical analytical framework of infrastructure digital transformation and urban carbon emissions, and on this basis, based on the panel data of 178 prefectural-level cities in China from 2005 to 2020, and using the "Smart City" pilot policy as a quasi-experiment, we empirically analyse the spatial spillover effect of infrastructure digital transformation on urban carbon emissions and its role by using the spatial difference-differences (SDID) model.

In view of this, the possible marginal contributions of this study are mainly in the following aspects: First, from the perspective of research, existing studies mainly focus on the impact of infrastructure on carbon emissions, and few studies have explored the relationship between infrastructure digital transformation and carbon emissions from the perspective of digital transformation of infrastructure. Secondly, this paper explores the impact effect of digital transformation from the macro-level urban infrastructure, and existing studies only start from the perspective of micro enterprises and industries, lacking macro-level research. Finally, this paper uses a SDID model to explore the spatial spillover effect of digital transformation of infrastructure on carbon emissions, answers the size of the spatial spillover range and regional boundaries, confirms the hypothesis of spatial distance attenuation, supplements the relevant studies on factors affecting urban low-carbon development, and provides new ideas on how to optimise the mechanism of inter-regional synergistic environmental governance in the next step.

The rest of this study is organised as follows. Section 2 reviews the literature. Section 3 introduces the policy context and formulates the research hypotheses. Section 4 presents the research methodology and data. Section 5 discusses the empirical results. Section 6 gives conclusions and policy implications.

## Literature review

With the growing popularity of sustainable urban development, a number of studies have been conducted on the impact of urban infrastructure on carbon emissions. Existing data and policies have verified that infrastructure affects urban carbon emissions. For one thing, each life stage of infrastructure involves a significant rise in carbon dioxide emissions results from the high energy consumption [10]. Some scholars have focused on traditional infrastructures such as "railroad and highway" transportation infrastructure and revealed that traditional transportation infrastructures contribute a lot to carbon emissions, and bring energy consumption, which can lead to high carbon emissions in nearby cities [11] as well as large amounts of carbon emissions in the local cities [12, 13]. Meanwhile, along with the continuous growth of high-speed rail network, high-speed rail can promote the aggregation of economic factors in the surrounding area, and facilitate the diffusion and spillover of innovations by facilitating the flowing of technology and knowledge in the city, thus reducing the neighbouring cities’ carbon emissions [14]. Representing the digital transformation of traditional transportation infrastructure, HSR can lower carbon emissions, which stems from the influence of industrial structure and technological innovation [15, 16].

For the other thing, digital technology, as embodied by big data, artificial intelligence and the Internet of Things promotes the birth of digital infrastructure, realizes the digital transformation and modernization of traditional infrastructure, provides intelligent technological means for the city’s social and economic development, promotes green technological innovation [17], and promotes the modernization of the industrial structure [18], thereby realizing the carbon emission reduction in all aspects of urban development. Digitalization is a new economic transformation drive that can transform the industrial structure of high labour intensity and high cost industries into high-tech content and environmentally friendly industries [19].

In addition, the replicability, non-exclusivity, and high mobility of digital technology, with modern information networks serving as important carriers, facilitate the transfer of information and knowledge among cities and have a significant spatial spillover effect on carbon emissions [20–23]. Digitalisation can help establish a coordinated regional carbon emission reduction model through spatial spillover effects [19]. To evaluate the environmental effects of urban infrastructure, some researchers have also employed quasi-natural experiments, such as "Smart City" pilot policy. As a new paradigm of modern urban governance in the modern era, smart city construction relies on artificial intelligence, cloud computing, and other digital technologies to facilitate the intelligent and digital development of traditional infrastructure including urban energy and transport, and to achieve the traditional infrastructure transformation and upgrading [24]. Therefore, from the concept and connotation of smart city, smart city construction can be seen as a significant embodiment of the digitalization of urban infrastructure. Using the "Broadband China" pilot policy [25, 26] and the "Smart City" pilot policy [27] as policy impacts on urban infrastructures’ digital transformation, they discovered that urban infrastructure significantly affects environmental governance. It is found that urban infrastructure plays an active role in environmental governance, helping to reduce carbon emissions in the local city and in neighbouring places.

Furthermore, the other type of literature relevant to this study is the economic environmental effects of digital transformation. The majority of current domestic and international research on digital transformation is concerned with how it affects economic development. The pattern of economic growth has been significantly impacted by digital transformation, thanks to the utilisation of cutting-edge technologies like artificial intelligence and big data, which were once popular topics of study for academics. Digital transformation has emerged as a key avenue for the superior growth of microenterprises in the age of the digital economy. Digital transformation may improve enterprise total factor productivity [28], enterprise innovation capacity [29], enterprise performance [30] and enterprise productivity [31], etc. It has been discovered that, at the industry level, digital transformation has spatial spillover effects on industrial structure upgrading [32] and industrial integration development [33]. Some scholars have also explored how the digital transformation is affecting the carbon emissions. Digital finance as a result of digital transformation helps to reduce the carbon intensity of Chinese cities with significant spatial spillover effects, which are more pronounced in cities with similar economic characteristics [34]. From a micro perspective, digital transformation can be a catalyst for green technological innovation in enterprises, which will undoubtedly have an impact on emission levels [35].

As previously stated, there are still certain shortcomings in the literature, even though it offers research ideas. Firstly, from the perspective of the research object, the existing research is limited to the impact of infrastructure on carbon emissions and lacks the effect of digital transformation of urban infrastructure on carbon emissions guaranteed by digital technology. Second, from the standpoint of the research, there is a dearth of research from the macro level of urban infrastructure in the literature of digital transformation, which is tightly linked to the research topic but mostly comes from the perspective of microenterprises and industries. Finally, the established literature ignores the issue of network externalities and lacks the spatial spillover effect of digital transformation of urban infrastructure on carbon emissions in terms of spatial relevance. Theoretically, during the procedure of digital transformation of urban infrastructure, relying on the network effect of digital technology, breaking the geographic segmentation restrictions, with network externalities. The application of the "Smart City" pilot policy has facilitated the open exchange of green technology innovation resources amongst cities and encouraged the transfer of green innovation expertise and other elements to nearby areas. Therefore, in analyzing the impact of the digital transformation of urban infrastructure on carbon emissions, it is essential to analyze the spatial spillover effect and spillover mechanism further. We consider the "Smart City" policy as the policy effect of digital transformation of urban infrastructure and analyzes the impact of digital transformation of urban infrastructure on carbon emissions from the "local-neighborhood" perspective.

## Policy background and theoretical mechanism analysis

### Policy background

Cities play a significant role as development hotspots in attaining the "dual carbon" goal. Digital transformation of infrastructure includes both the convergence of digital technologies with traditional infrastructure and the emergence of new types of infrastructure, such as digital infrastructure. Smart cities are a crucial component of advancing the digital transformation of cities and an important example of the digital transformation of infrastructure in the new era. China began implementing its "Smart City" pilot policy at the end of December 2012, with the first batch of pilot cities involving a total of 90 prefectural and county-level cities. Several batches of pilots followed in 2013 and 2015. By the end of 2015, there are nearly 300 pilot cities. It is foreseeable that the "Smart City" pilot policy will have a significant and far-reaching impact on all aspects of China’s economic development. "Smart City" pilot cities are slightly more digitised than non-pilot cities, which is directly related to the development of the city’s digital economy. "Smart City" construction is an outward expression of an urban infrastructure’s digital transformation and is consistent with the idea that the digital economy can sustain urban development. Therefore, "Smart City" pilot cities have enhanced the level of urban digitization and accelerated the process of digital transformation of urban infrastructures. This has had a major effect on urban carbon emission reduction and performance enhancement.

### Direct impact mechanism

The digital transformation of infrastructure is a necessary precondition for fostering the in-depth development of the digital economy and is essential to enhancing the digital economic environment. As a critical technology for the industrial and technological revolutions, digital technology will be crucial in tackling climate change and creating a wealth of opportunities towards low-carbon development.

Specifically, the application of cutting-edge digital technologies like big data, blockchain, artificial intelligence, and others empowers traditional urban infrastructure, realizing the digital transformation of traditional infrastructure, which is widely used in the fields of industrial development and life services and significantly alters human production and way of life [36]. First of all, he public can now shop online and work from paperless offices thanks to the digital transformation of infrastructure, which has also led to the establishment of several Internet industries and platforms. This has greatly reduced energy usage and carbon emissions from manufacturing and shipping. Furthermore, the networked nature of the digital transformation of infrastructure has facilitated the expansion of the scale of Internet users, which is conducive to the rapid penetration of information and knowledge. It has also been crucial in encouraging people to adopt greener and lower-carbon consumption practices, raising public awareness of environmental issues, and cutting carbon emissions. Secondly, digital technology acts on traditional industries, providing intelligent equipment and production management modes that support green production methods and encouraging the modernization and transformation of traditional industries to mitigate carbon emissions [37]. Reduced energy consumption in the production process, more environmentally friendly features, and a decrease in adverse environmental effects are the results of digital transformation, which encourages the conversion of traditional manufacturing industries to smart manufacturing and develops green and intelligent production methods [38]. Finally, the digital transformation of infrastructure also enables real-time pollution monitoring, lowers government regulatory costs, and offers intelligent technical support for environmental governance and supervision by the government to minimize carbon emissions. enables real-time pollution monitoring, lowers government regulatory costs, and offers intelligent technical support for environmental governance and supervision by the government to minimize carbon emissions. On the basis of the above analyses, the study proposes the following hypotheses:

Hypothesis 1: Digital transformation of urban infrastructure reduces local carbon emissions.

### Indirect impact mechanism

The contribution of industrial structure optimization and upgrading in boosting carbon emission reduction has been generally verified [39, 40]. Overall, the digital transformation of urban infrastructure serves an important function in fostering the growth and development of the digital economy. This has resulted in the emergence of a new wave of information technology industries encompassing big data, mobile Internet, cloud computing, Internet of Things, and more, challenging established energy-dependent industries [41] and pressuring more established, high-pollution, high-energy-consumption industries to be eliminated, transformed, and upgraded [42]. Meanwhile, data is a clean and effective production factor that can reduce the reliance on and use of natural resources and help traditional enterprises undergo a digital transformation [43]. Digital transformation is crucial to improving energy consumption and reducing carbon emissions. For one thing, digital transformation optimizes the production mode promotes the industrial structure’s gradual transformation from being dominated by heavy industry to being dominated by the service industry, so as to achieve the optimisation of the industrial structure [44]. For the other thing, the digital technology development of digital technology can facilitate the transfer of resource factors from low-end to high-end services. Increasing the proportion of service industries in the overall industrial structure will help to reduce air pollution [45]. Furthermore, the digital industry’s strategic application of elements like 5G and artificial intelligence fosters the development of new industries that align with current development concepts. This, in turn, supports the transformation of the industrial structure into forms that are environmentally friendly and technology-intensive [46], as well as the reduction of carbon emissions associated with urban areas.

Technological innovation, especially green technology innovation, is seen as a key strategy for lowering carbon emissions [47, 48]. With data serving as the primary production factor and possessing both technological and green attributes, the digital transformation of urban infrastructure has the potential to unleash inexhaustible power for low-carbon urban development by promoting green technological progress. Digitalization will promote technological innovation in all aspects of production [49], promote the broad application of green low-carbon technologies and energy-saving equipment, which will also tend to force the energy consumption structure to shift to a mode that is more energy-efficient and consumes less energy, lessen reliance on fossil fuels, and increase energy use efficiency to lower urban carbon emissions [50]. In addition, digital technologies such as information networks with inter-temporal characteristics have reduced the cost of information transfer, realized instant communication between regions, and accelerated the interaction of information and the dissemination of knowledge. The information flow facilitates knowledge spillovers, strengthens the interregional diffusion of green technologies and improves the regional green technology innovation level, thus reducing intra-regional carbon emissions. Given the conversation above, we propose Hypothesis 2.

Hypothesis 2: By encouraging the optimization of the urban industrial structure and the development of green technologies, the digital transformation of urban infrastructure lowers carbon emissions from cities.

### Spatial spillover effect

Digital technologies such as information networks with inter-temporal characteristics have reduced the cost of information transmission, realized instant communication between regions, and accelerated the interaction of information and the dissemination of knowledge. Due to the network and information characteristics, the digital transformation of urban infrastructure breaks the spatial and geographical restrictions, strengthens the geospatial connection between cities, promotes the sharing and flow of knowledge elements, data elements, and other production factors between cities, makes different industries share resources and technologies, influences neighboring cities’ carbon emissions through the spread of technological and knowledge spillover, and results in clear urban environmental governance spatial spillover effect [22]. This effect will diminish as the information decays during spatial transmission, which means that the spatial spillover effects of digital transformation of urban infrastructure will have certain regional boundaries. Specifically, the digital transformation of urban infrastructure can lower nearby cities’ the carbon emission intensity by "demonstration effect". The center-edge city theory states that as digital technology advances, intangible knowledge such as emission reduction technology, green development experience, and local systems may spread and diffuse, reducing carbon emissions in nearby regions [51]. Meanwhile, local governments have imitative behaviors in formulating and implementing policies [52]. The digital transformation of urban infrastructure can increase carbon emissions in nearby cities through the "siphon effect". Driven by emerging digital technologies, the digital infrastructure transformation may result in stricter environmental regulations in local cities, resulting in highly polluting industries being forced to relocate to neighboring cities, thus raising nearby cities’ carbon emissions. This is comparable to the well-known "pollution haven" theory, which cities with laxer environmental laws have been the preferred location for pollution-intensive businesses. Furthermore, because of the region’s limited resources, the smart pilot cities typically receive the resources and other components required for low-carbon and green development, thus discouraging the neighbouring cities’ green development. Due to the analysis above, this study puts forward the following hypothesis 3:

Hypothesis 3: Digital transformation of urban infrastructure has a spatial spillover effect and affects nearby cities’ carbon emissions.

**Fig 1:**
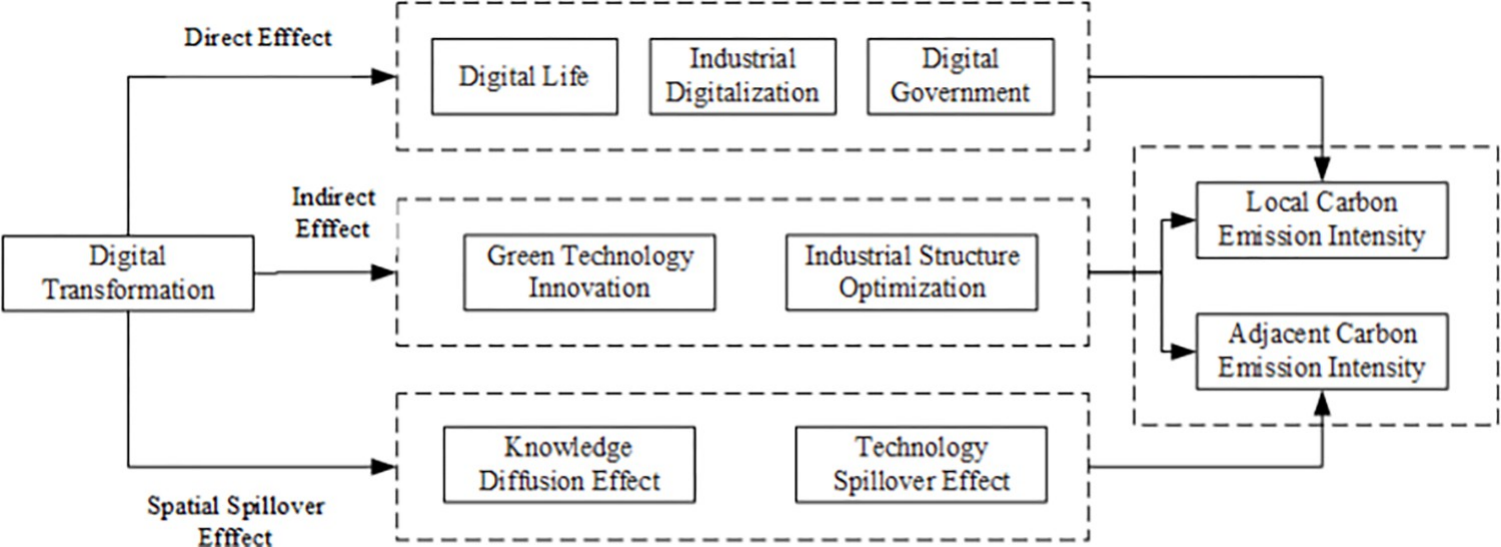
The theoretical framework for the effect of digital economy on carbon emission intensity.

The theoretical framework is shown in Fig 1.

## Methodology and data

### Benchmark model

Based on the previous theoretical analysis, neighboring cities are impacted by the digital transformation of urban infrastructure in addition to local carbon emissions. Therefore, this study builds the spatial difference-in-differences (SDID) model on the baseline of the difference-in-differences (DID) model:

$$LNCEI_{\mathrm{it}}=\rho WLNCEI_{it}+\alpha_{1}D_{\mathrm{it}}+\alpha_{2}WD_{it}+\beta_{1}X_{it}+\beta_{2}WX_{it}+\mu_{i}+\nu_{t}+{\left(1-\lambda W\right)}^{-1}\varepsilon_{it}$$ (1)

Where, *i* dentifies the city, *t* identifies the year. *LNCEI*_it_ is the dependent variable used in this paper, representing the carbon emission intensity. *X*_it_ represents the control variables, *D*_*it*_ is the dummy variable indicating that if the city i has implemented the "Smart City" pilot policy in year t, then *D*_*it*_ = 1, otherwise, *D*_*it*_ = 0. *μ*_*i*_ represents the spatial-fixed effects; *ν*_t_ represents the time-fixed effects; *ε*_it_ is the random error term. W is the spatial weight matrix, In this paper, we use the geographic distance weight matrix, that is, the reciprocal of the square of the geographic distance between two cities is chosen to construct the matrix.

Second, following the previous analysis, the digital transformation of urban infrastructure may influence carbon emissions via industrial structure optimization and green technological innovation. Thus, referring to the mediation effect model of Baron and Kenny [53], we created the following model based on SDM-DID, as shown in (2) and (3):

$$M_{\mathrm{it}}=\rho WLNCEI_{it}+\alpha_{1}D_{\mathrm{it}}+\alpha_{2}WD_{it}+\beta_{1}X_{it}+\beta_{2}WX_{it}+\mu_{i}+\nu_{t}+\varepsilon_{it}$$ (2)

$$LNCEI_{\mathrm{it}}=\rho WLNCEI_{it}+\alpha_{1}D_{\mathrm{it}}+\alpha_{2}WD_{it}+\beta_{1}X_{it}+\beta_{2}WX_{it}+\gamma M_{\mathrm{it}}+\mu_{i}+\nu_{t}+\varepsilon_{it}$$ (3)

In Eqs (2) and (3), *M*_*it*_ is the mechanism variable, which represents the two transmission pathways of industrial structure optimization and green technological innovation. *γ* represents the impact of the digital transformation of urban infrastructure on carbon emissions after adding the mechanism variable.

### Variables and data

#### Dependent variable

The dependent variable *CEI*_it_ is carbon emission intensity, measured using CO_2_ emissions per unit of GDP [54].

#### Core explanatory variable

We use exogenous shocks from the implementation of the "Smart City" pilot policy (*D*_*it*_) as a proxy variable for the digital transformation of urban infrastructure. If city *i* is a smart city pilot at time t, *D*_*it*_ equals 1, otherwise 0. Since the first smart city pilots were established in December 2012, this study treats 2013 as the first effective year for this policy. After deleting data missing from the sample, we selected 178 prefecture-level cities, including 31 cities in the treatment group and 147 in the control one.

#### Control variables

The control variables are as follows: economic development level (Pgdp), measured by the city’s per capita GDP; foreign direct investment (Fdi), measured by the ratio of the total amount of foreign capital used to the GDP of each city after conversion of the exchange rate in the current year; urbanization level (Urb), measured by the ratio of the city’s resident population to the total population; traditional infrastructure level (Tra), measured by urban road area per capita; information infrastructure level (Inf), measured by the number of Internet access households per 10,000 people.

#### Mechanism variables

The mediating variables are industrial structure optimization (Instr) and green technological innovation (Gti). Industrial structure optimization (Instr) is measured by the ratio of the output value of the tertiary industry to GDP. Green technological innovation (Gti) is represented by the number of green patents granted.

#### Data source and description

We have utilized data from 178 China prefecture-level cities between 2005 and 2020 as our research subject. Since the data on CO_2_ emissions for some prefecture-level cities were missing, we calculated the total CO_2_ emissions of each prefecture-level city by summing up the inferred district and county CO_2_ emissions data from the CEADs database. Green patents were obtained through the China Research Data Service Platform (CNRDS). Other raw data for this study were obtained from the China Statistical Yearbook, China Urban Statistical Yearbook, and China Regional Economic Statistical Yearbook. Some prefecture-level cities have a slight missing data problem, which is filled by consulting each province’s and city’s statistical yearbooks, each city’s annual statistical bulletin, the interpolation method, the annual average growth rate, and other ways. All data related to price factors are indexed by the price index (2005 = 100) to cancel the effect of price factors. Prior to the empirical tests, the variables were all logarithmised to eliminate the effects caused by heteroskedasticity. The number of green patents granted to all prefecture-level cities in each year is added to 1 before taking the logarithm of the green technological innovation (Gti). The above variables’ descriptive statistical results are displayed in Table 1.

**Table 1:** Descriptive statistics for major variables.

Variable Category	Variable Name	Mean	Std. Dev.	Min	Max	Obs
Explained variable	*LnCEI*	7.632	0.596	4.388	10.513	2,848
Core explanatory variable	*D*	0.098	0.297	0	1	2,848
Control variables	*LnPgdp*	10.225	0.728	8.059	12.323	2,848
	*LnFdi*	-4.515	1.308	-10.414	-1.257	2,848
	*LnUrb*	3.890	0.332	2.633	4.605	2,848
	*LnTra*	2.336	0.599	-2.526	4.272	2,848
	*LnInf*	3.840	1.138	-3.912	6.785	2,848
Intermediary variables	*LnGti*	2.434	1.779	0	7.601	2,848
	*LnInstr*	3.642	0.249	2.402	4.350	2,848

## Empirical analysis

### Spatial autocorrelation analysis

Before using the spatial model to examine the spatial effect of digital transformation of urban infrastructure on urban CEI, the spatial correlation of CEI across cities needs to be examined to observe the spatial distribution characteristics. Here, we use the global Moran’s I to judge it. The calculation model is as follows.


$$I=\frac{n\sum_{i=1}^{n}\sum_{j=1}^{n}W_{ij}(y_{i}-\bar{y})(y_{j}-\bar{y})}{\sum_{i=1}^{n}\sum_{j=1}^{n}W_{ij}{\left(y_{i}-\bar{y}\right)}^{2}}$$ (4)

Where, *I* is the global Moran’s I value, *n* represents 178 cities, *y*_*i*_ and *y*_*j*_ represent the carbon emission intensity values of cities i and j, respectively. $\bar{y}$ refers to the mean value of carbon emission intensity of the 178 cities. The value of Moran index is in the range of [–1,1]. If the Moran index is negative, it means that the corresponding variables are distributed in the second and fourth quadrants, showing the spatial clustering characteristics of high—low or low—high; if the index is positive, it means that the corresponding variables are distributed in the first and third quadrants, showing the spatial clustering characteristics of high—high or low—low. The closer the score is to 0, the weaker the spatial correlation of the corresponding variable is.

We use the geographical distance weight matrix to calculate the global Moran’s index from 2005 to 2020. The results of the calculations are shown in Table 2. As Table 2 demonstrates, the Moran’ s index is significantly positive at 1%, indicating that CEI have significant high (low)–high (low) spatial agglomeration characteristics—that is, areas with high (low) values are surrounded by areas with high (low) values, and both exhibit spatial spillover. The spatial correlation between CEI has generally increased; specifically, the Moran’ s index of CEI fluctuates repeatedly within the 16-year period. This spatial correlation may be attributable to geographical and economic factors and the spatial and temporal differences of urbanization level.

**Table 2:** Moran’s index of main variables.

year	W1	P value	W2	P value	W3	P value
2005	0.121	0.000	0.287	0.000	0.148	0.000
2006	0.124	0.000	0.293	0.000	0.151	0.000
2007	0.128	0.000	0.299	0.000	0.155	0.000
2008	0.130	0.000	0.302	0.000	0.157	0.000
2009	0.132	0.000	0.308	0.000	0.157	0.000
2010	0.133	0.000	0.311	0.000	0.159	0.000
2011	0.121	0.000	0.290	0.000	0.148	0.000
2012	0.121	0.000	0.290	0.000	0.147	0.000
2013	0.123	0.000	0.302	0.000	0.148	0.000
2014	0.124	0.000	0.302	0.000	0.148	0.000
2015	0.134	0.000	0.317	0.000	0.158	0.000
2016	0.140	0.000	0.327	0.000	0.163	0.000
2017	0.134	0.000	0.317	0.000	0.160	0.000
2018	0.132	0.000	0.315	0.000	0.159	0.000
2019	0.133	0.000	0.320	0.000	0.161	0.000
2020	0.134	0.000	0.325	0.000	0.161	0.000

## Parallel trend test

The underlying presumption in utilising the SDID model is that a parallel trend exists between the control and treatment groups prior to policy implementation that does not change over time [55]. In this regard, this paper adopts the event study method to further test this by establishing the following model (5):

$$LNCEI_{\mathrm{it}}=\alpha_{0}+\sum_{y=1}^{6}\alpha_{\mathrm{pre}\_1}D_{\mathrm{pre}\_1}+\alpha_{\mathrm{current}}D_{\mathrm{current}}+\sum_{y=1}^{2}\alpha_{\mathrm{post}\_1}D_{\mathrm{post}\_1}+\sum \alpha_{i}{\mathrm{control}}_{it}+\mu_{i}+\nu_{t}+\varepsilon_{it}$$ (5)

**Fig 2:**
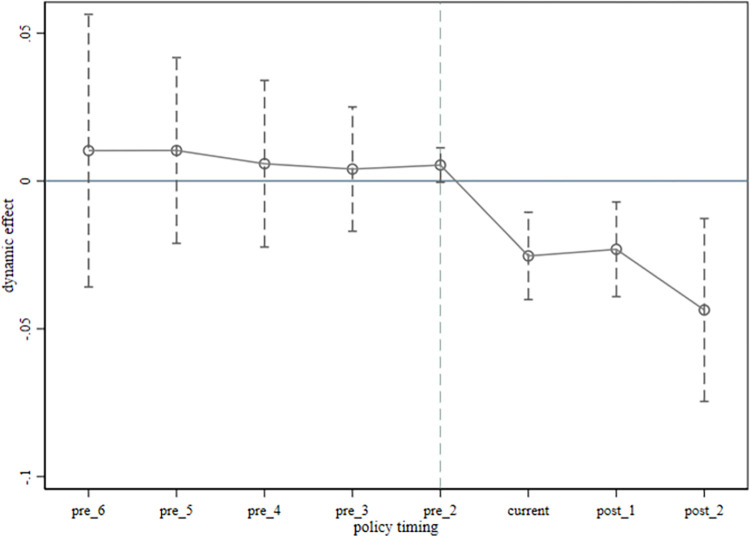
Parallel trend test results.

Where, Dpre_1、Dcurrent、Dpost_1 are the cross-multipliers of the year dummy and the policy dummy for the year before, the year of and the year after the implementation of the "smart city" policy, respectively; αpre_1、αcurrent、αpost_1 are the coefficients of the corresponding coefficients, and the significance is the same as that of the previous section. The regression results are shown in Fig 2.

From Fig 2, it can be seen that the coefficients of the experimental group and the control group in the six years before the implementation of the policy are not significant, and the confidence intervals contain 0 value, which indicates that before the implementation of the "Smart City" policy, the carbon emissions of the experimental group and the control group maintain the same trend of change, there is no significant difference, which is in line with the hypothesis of parallel trend test. However, in the year of policy implementation and two years after the implementation of the policy, the regression coefficients are significantly negative, and the confidence interval does not contain 0 value, which indicates that under the impact of the policy, the carbon emissions of the experimental group and the control group appear to be different, and the trend of carbon emission reduction of the two groups appears to be obvious, and the parallel trend in the past is broken. Therefore, the parallel trend test of this paper is valid, which means that the results of the benchmark regression using the SDID model in this paper are valid.

### Spatial model selection test

To select a suitable global spatial econometric model, a series of standard spatial model selection tests were conducted. First, the LM test shows that both the LM and robust LM values of the SAR and SEM models are significant, which implies that the spatial econometric model should be selected. To further test whether SDM degenerates into SEM or SAR, Wald test and LR test were then conducted respectively. The spatial Hausman test was then performed and the model was finally determined to be a two-way fixed SDM. The test results of the model are shown in Table 3.

**Table 3:** Results of spatial model selection test.

Tests	Statistic	Tests	Statistic
LM-lag	1468.676***	LR-spatial-lag	78.32***
Robust LM-lag	6.822***	LR-spatial-error	68.48***
LM-error	2376.657***	Wald-spatial-lag	12.91**
Robust LM-error	914.804***	Wald-spatial-error	13.40**
Hausman检验	16.455******		

Standard errors in parentheses

*** p<0.01

** p<0.05

* p<0.1

### Results of the SDID model

Table 4 displays the empirical results of how urban infrastructure’s digital transformation has affected carbon emissions. For comparison, Table 4 also presents the DID, SLM-DID, SEM-DID, and SDM-DID regression results. The results of all models demonstrate that the "Smart City" pilot policy’s coefficient is noticeably negative. According to the results of all models, we can conclude that the "Smart City" pilot policy shows a significantly negative coefficient. This suggests that the digital transformation of urban infrastructure lowers local cities’ carbon emissions, supporting Hypothesis 1. Furthermore, the coefficients of DID model for the "Smart City" pilot policy are more significant than the results of any SDID model, suggesting that, if spatial spillover effects are disregarded, the influence of digitally transforming urban infrastructure on carbon emissions is overstated.

**Table 4:** The benchmark regression results.

	(1) DID	(2) DID	(3) SAR-DID	(4) SEM-DID	(5) SDM-DID
Variable	CEI	CEI	CEI	CEI	CEI
*D*_*it*_	-0.046**	-0.044**	-0.027***	-0.018***	-0.023***
	(0.018)	(0.017)	(0.006)	(0.006)	(0.006)
*LNPgdp*		-0.092	-0.031**	0.032	0.139***
		(0.060)	(0.015)	(0.025)	(0.030)
*LNFdi*		-0.006*	-0.002	0.0002	0.001
		(0.003)	(0.001)	(0.002)	(0.002)
*LNUrb*		0.065**	0.074***	0.096***	0.097***
		(0.028)	(0.009)	(0.010)	(0.010)
*LNTra*		0.005	0.009**	0.012***	0.010**
		(0.009)	(0.004)	(0.004)	(0.004)
*LNInf*		-0.002	0.001	0.002	0.003
		(0.009)	(0.003)	(0.003)	(0.003)
cons	8.218***	8.845***			
	(0.011)	(0.575)			
TW* *D*_*it*_					-0.072***
					(0.019)
TW* *LNPgdp*					-0.294***
					(0.054)
TW* *LNFdi*					-0.007*
					(0.004)
TW* *LNUrb*					-0.127***
					(0.026)
TW* *LNTra*					-0.045***
					(0.014)
TW* *LNInf*_*t*_					0.003
					(0.010)
rho			0.856***	0.878***	0.824***
			(0.022)	(0.020)	(0.024)
sigma2_e			0.003***	0.003***	0.003***
			(0.000)	(0.000)	(0.000)
N	2848	2848	2848	2848	2848
R^2^	0.987	0.987	0.006	0.236	0.107

Standard errors in parentheses

*** p<0.01

** p<0.05

* p<0.1

The spatial correlation coefficient (rho) at the 1% level is significantly positive according to the regression results of SDM-DID, which indicates that the digital transformation of urban infrastructure exerts a spatial spillover effect on carbon emissions. Following the partial differentiation method’s decomposition of the spatial effect, Table 5 displays the outcomes.

**Table 5:** The estimated results of the direct, indirect, and total effects.

	(1) SDM	(2) Direct effect	(3) Indirect effect	(4) Total effect
Variable	LNCEI	LNCEI	LNCEI	LNCEI
*D*_*it*_	-0.023***	-0.033***	-0.507***	-0.540***
	(0.006)	(0.006)	(0.109)	(0.111)
W* *D*_*it*_	-0.072***			
	(0.019)			
rho	0.824***			
	(0.024)			
Control Variables	YES	YES	YES	YES
Spatial fixed effects	YES	YES	YES	YES
Time fixed effects	YES	YES	YES	YES
N	2848	2848	2848	2848
R^2^	0.107	0.107	0.107	0.107

Standard errors in parentheses

*** p<0.01

** p<0.05

* p<0.1

As shown in Table 5, the direct and indirect effects of the digital transformation of urban infrastructure are clearly significant at the 1% level, implying the digital transformation of urban infrastructure has significant "local-neighborhood" synergistic effects, which greatly lower carbon emissions in the neighborhood. This is confirmed that there is a spatial spillover effect from the digital transformation of urban infrastructure, verified hypothesis 2. This result may be because urban infrastructure relies on the network effect of information technology during digital transformation, and there is the phenomenon of environmental "free-riding" between neighboring regions, which promotes the trans-regional circulation of talents and technologies through the knowledge spillover effect, so that the local green technologies can play a role in the neighboring regions, bringing environmental development to the neighboring regions. The knowledge spillover effect promotes the cross-regional circulation of talents and technologies, enabling local green technologies to play a role in neighboring regions, bringing positive impacts on the environmental development of neighboring regions, reducing carbon emissions in nearby areas, and resulting in a situation where carbon emissions are reduced for both "local and neighboring regions" in a mutually beneficial way.

### Regional boundary

According to geography’s spatial first law hypothesis, the spatial dependence or spillover effect steadily diminishes as interregional distance rises. This means the spatial spillover effects of the digital transformation of urban infrastructure on carbon emissions may be more significant within a certain spatial range. In contrast, outside of that range, the effects may diminish or disappear due to distance decay effects. As such, the digital economy’s spillover effect on CEI is limited to a specific spatial scale, that is, the regional boundary.

This paper sets different spatial distance thresholds to estimate the regional boundary based on the spatial weight matrix of geographic distances W, which is shown in Eq (6):

$$W_{\mathrm{ij}}=\{\begin{array}{l} \frac{1}{d_{ij}},d_{\mathrm{ij}}\geq d\\ 0,\;d_{\mathrm{ij}}<d \end{array}$$ (6)

Where, d_ij_ is the distance between city i and city j, d is the distance threshold. We set the initial distance threshold at 100 km and then incremented it in 100 km increments up to 600 km and then regressed it using SDM-DID.

**Fig 3:**
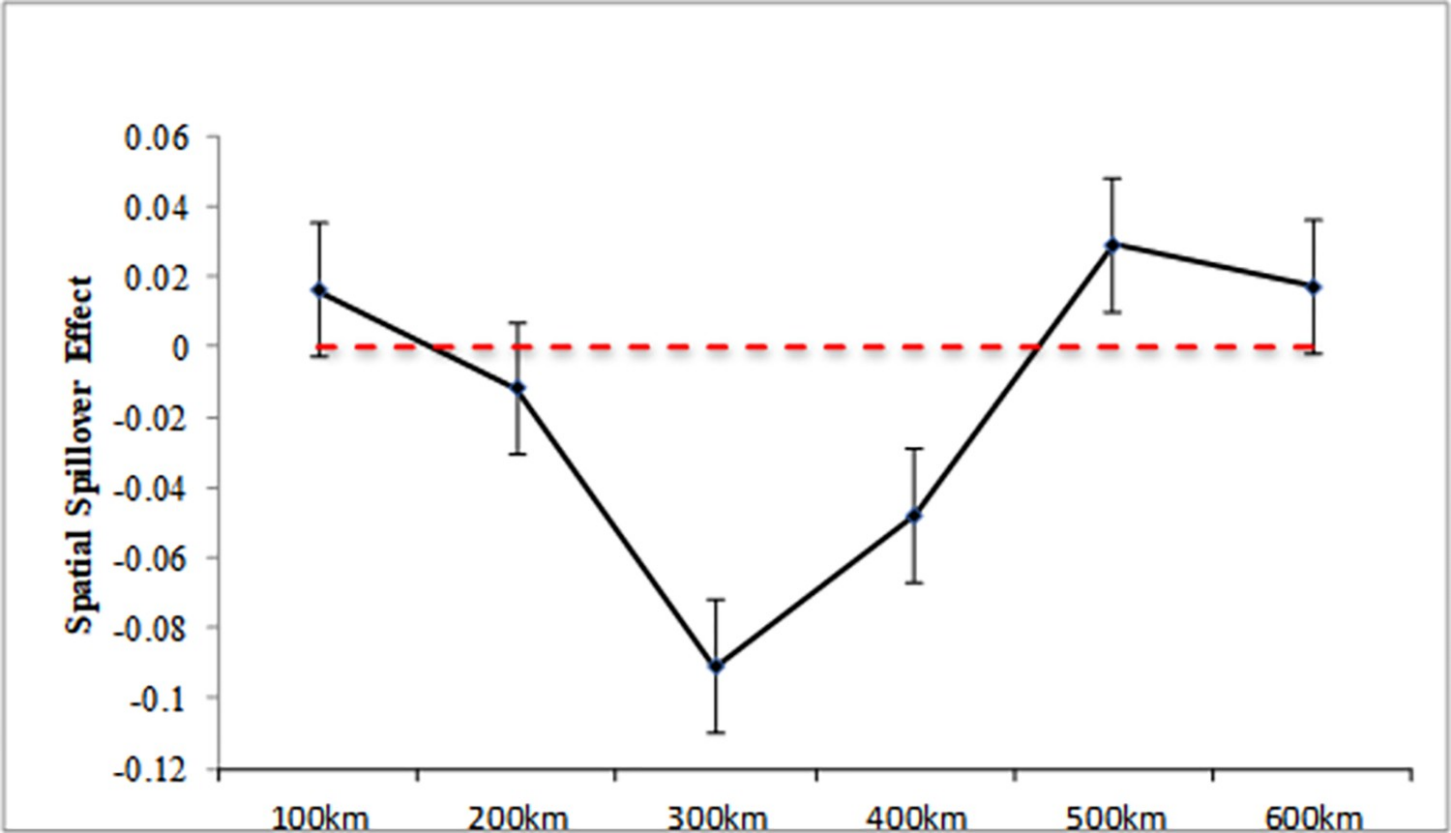
Spatial attenuation boundary.

From Fig 3, it can be shown that the impact of digital transformation of urban infrastructure on carbon emissions intensity in neighbouring cities is significant within 600km, indicating that the spatial spillover of urban infrastructure’s digital transformation has a regional boundary on carbon emissions intensity. Specifically, the digital transformation of urban infrastructure has a substantially negative spatial spillover coefficient within 500 km, which exhibits a U-shaped trend and peaks at 300 km at -0.091. This suggests that within that range, the urban infrastructure digital transformation can have a "demonstration effect" that greatly lower carbon emissions in nearby cities. Geographic proximity facilitates the dissemination of knowledge, systems, and green and low-carbon technologies, which are mutually beneficial to neighboring regions and lead to the low- and green-carbon development of neighboring cities. After 500 km, the spatial spillover effect is positive, meaning that the digital transformation of urban infrastructure in this range can increase neighbouring cities’ carbon emissions by absorbing their green resources and factors. After 600 km, the spatial spillover effect coefficient is not significant, suggesting that there is a specific spatial spillover boundary for the influence of digital transformation of urban infrastructure on nearby areas’ carbon emissions.

### Mechanism analysis

This paper further combines the SDM-DID model to explore whether urban carbon emissions can be impacted by the digital transformation of urban infrastructure through the two mechanisms of green technological innovation and industrial structure optimization. Table 6 demonstrates the regression results.

**Table 6:** The impact mechanisms of smart city construction on CEI.

		(1)	(2)	(3)	(4)	(5)
	Variable	LNCEI	LNGti	LNCEI	LNInstr	LNCEI
Direct effect	*D*_*it*_	-0.033***	0.187***	-0.025***	0.003	-0.035***
		(0.006)	(0.051)	(0.007)	(0.009)	(0.007)
	*LNGti*			-0.008***		
				(0.002)		
	*LNInstr*					0.039***
						(0.012)
Indirect effect	*D*_*it*_	-0.507***	2.650***	-0.279***	0.238***	-0.546***
		(0.109)	(0.357)	(0.105)	(0.072)	(0.130)
	*LNGti*			-0.130***		
				(0.028)		
	*LNInstr*					0.379**
						(0.152)
Total effect	*D*_*it*_	-0.540***	2.837***	-0.305***	0.241***	-0.581***
		(0.111)	(0.361)	(0.109)	(0.073)	(0.133)
	*LNGti*			-0.138***		
				(0.028)		
	*LNInstr*					0.417***
						(0.154)
	rho	0.824***	0.530***	0.796***	0.581***	0.812***
		(0.024)	(0.035)	(0.026)	(0.033)	(0.025)
	Control Variables	YES	YES	YES	YES	YES
	Spatial fixed effects	YES	YES	YES	YES	YES
	Time fixed effects	YES	YES	YES	YES	YES
	N	2848	2848	2848	2848	2848
	R	0.107	0.140	0.122	0.010	0.079

Standard errors in parentheses

*** p<0.01

** p<0.05

* p<0.1

Column (1) demonstrates that the digital transformation of urban infrastructure significantly reduces urban carbon emissions. Column (2) demonstrates that the direct, indirect, and total effects of urban infrastructure digital transformation on green technology innovation are all significantly positive. This suggests that the digital transformation of local urban infrastructure not only promotes local green technology innovation but also the green technology innovation of neighboring cities, thus indicating that there is a spillover effect of the digital transformation of local urban infrastructure, which can produce positive green spillover effects on neighboring cities. Column (3) simultaneously incorporates the digital transformation of urban infrastructure and green technology innovation in the regression model. The results demonstrate that the coefficient of green technology innovation in the direct effect decreases compared with that in the model without mediating variables, but the significance of the coefficient remains unchanged. This suggests the existence of a partial mediating effect. The digital transformation of local urban infrastructure lowers carbon emissions of the local city through green technology innovation. Furthermore, the indirect effect demonstrates that green technology innovation in nearby areas lowers carbon emissions in those areas as well as acting as a mechanism for local urban infrastructure innovation and carbon emission reduction in nearby areas.

The results in column (4) demonstrate that the digital transformation of local urban infrastructure has a significantly positive coefficient of indirect effect on the optimization of local industrial structure, indicating that the digital transformation of local urban infrastructure facilitates the realization of industrial structure optimization in nearby places. Column (5) incorporates both digital transformation of urban infrastructure and industrial structure optimization into the regression model. The direct effect is significantly positive, meaning the optimization of industrial structure in neighboring places increases urban carbon emissions. The results indicate that the digital transformation of local urban infrastructure will have a certain optimisation effect on the industrial structure of the nearby cities, which in turn will have a spatial spillover effect on the carbon emissions of the neighbouring cities. It confirms part of hypothesis 3 of this study.

### Robustness test

**Fig 4:**
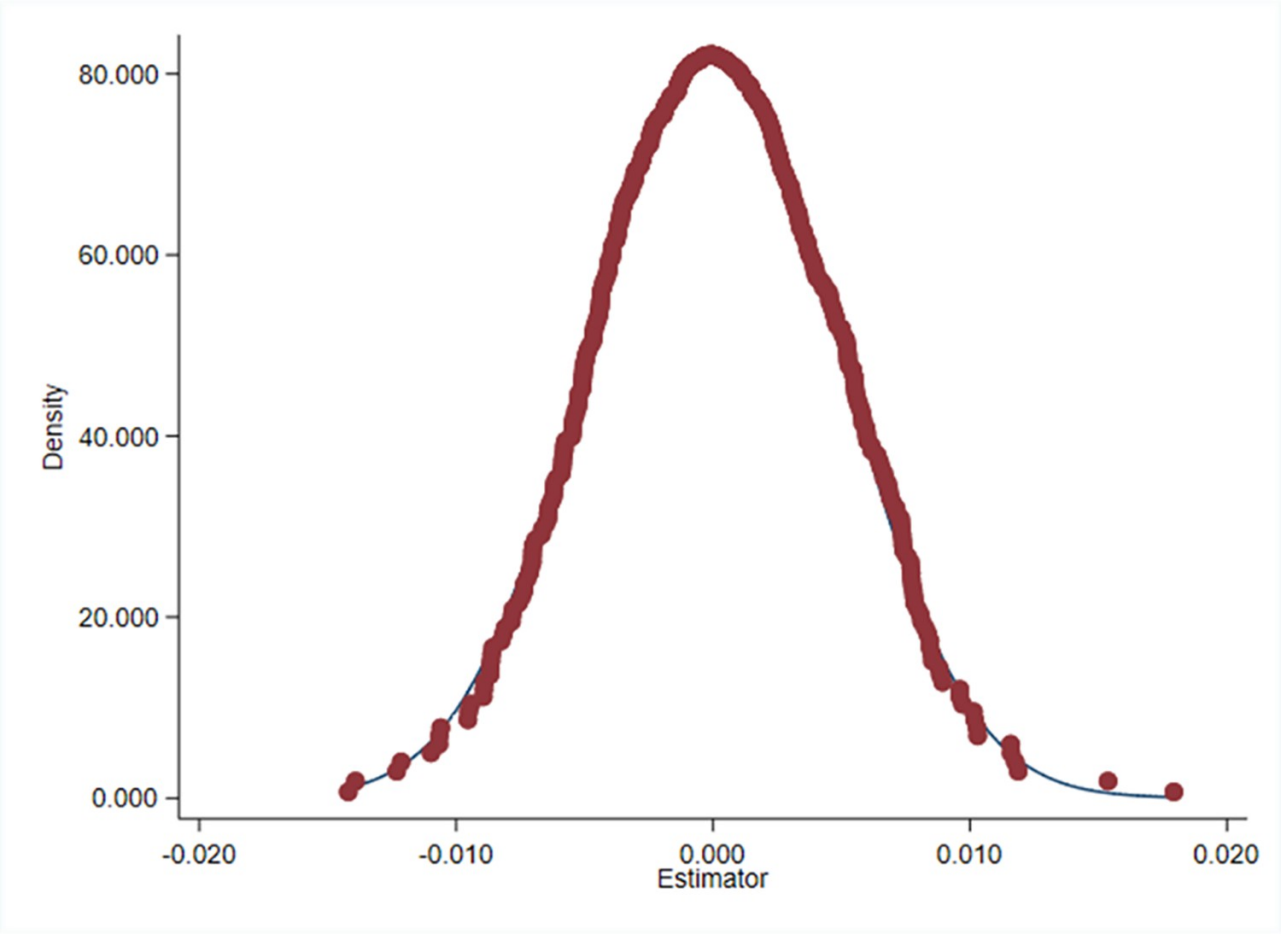
Placebo test.

We ran a robustness test to confirm the conclusions’ dependability even more. First, the provincial capital cities are excluded. As a result of the superiority of provincial capital cities over other prefecture-level cities in terms of resource agglomeration and economic development, this paper eliminates all of the provincial capital city data from the sample. The results, as shown in the column (1) of Table 7, show that the coefficients continue to be significant and consistent with the baseline regression’s results. Second, the results in column (2) of Table 7 show that the sign and significance level of the coefficients are in line with the baseline results when the second and third pilot cities are added to the sample to form a new treatment group. Third, the spatial weighting matrix is replaced and the robustness of the previous spatial measure estimation results is tested by comparing the estimation results of different spatial weighting matrices. The geographic distance weight matrix and geographic neighborhood 0–1 matrix are used to re-run the regression, and the test results are shown in columns (3) and (4) of Table 7, demonstrating that the test results’ coefficients are still significant and in line with the findings of the benchmark regression. Finally, this paper conducted 500 randomized tests on 178 sample cities, and the results are displayed in Fig 4, where the robustness of the benchmark regression results is confirmed by the kernel density plots of the regression coefficients following a normal distribution.

**Table 7:** The regression results of the robustness checks.

	(1)	(2)	(3)	(4)
Direct effect	-0.033***	-0.026***	-0.064***	-0.040***
	(0.006)	(0.005)	(0.010)	(0.007)
Indirect effect	-0.398***	-0.269***	-5.882***	-1.159***
	(0.080)	(0.091)	(1.419)	(0.227)
Total effect	-0.432***	-0.295***	-5.946***	-1.199***
	(0.082)	(0.093)	(1.427)	(0.228)
rho	0.765***	0.803***	0.890***	0.492***
	(0.028)	(0.027)	(0.027)	(0.087)
Control Variables	YES	YES	YES	YES
Spatial fixed effects	YES	YES	YES	YES
Time fixed effects	YES	YES	YES	YES
N	2576	3280	2848	2848
R^2^	0.110	0.004	0.160	0.235

Standard errors in parentheses

*** p<0.01

** p<0.05

* p<0.1

## Conclusions and policy recommendations

Based on the "local-neighbourhood" perspective, using panel data from 178 Chinese prefecture-level cities from 2005 to 2020 and the "Smart City" pilot policy as a quasi-natural experiment, this study investigates the impact of the digital transformation of urban infrastructures on carbon emissions. The results demonstrate that the digital transformation of urban infrastructure has a significant reduction in the local cities’ carbon emission intensity while also generating a spatial spillover effect that lowers the nearby cities’ carbon emission intensity, and that this spatial spillover effect has a regional boundary of 600 kilometres. After several robustness tests, the results still hold. The mechanism test shows the digital transformation of urban infrastructure is able to drive green technological innovation in local and neighboring places to achieve the synergistic carbon emission reduction in "local-neighboring" cities, whereas the digital transformation of local urban infrastructure contributes to the optimization of the industrial structure of neighboring places, but increases the local cities’ carbon emission intensity.

Based on the aforementioned findings, this study presents the following policy insights. First and foremost, the government need deeply grasp the spatial spillover effect of urban infrastructure digitization, enhance the digital economy’s enabling role, direct the digital economy’s flow and diffusion to underdeveloped areas, and actively support the development of "Smart city" as a pilot project. Furthermore, digitizing urban infrastructure has a spatial spillover effect on carbon emissions from cities that is regional in nature, decays with time, and is impeded by provincial boundaries. This necessitates local governments to foster greater inter-regional exchange, facilitate cross-regional digital technology flow through enhanced collaboration, and promote synergistic development across regions. Efforts should focus on minimizing the spatial reach of urban infrastructure digitization’s impacts while maximizing positive spillover effects. Finally, the government ought to fully release the innovation dividend brought by the knowledge spillover effect. Policymakers should strengthen investment in green technology innovation, promote it in production, and encourage the integration of new-generation information technology with traditional infrastructure. Policymakers should also accelerate the establishment of new industries that are sustainable, recyclable, and develop a mechanism through which the industrial structure aids in the reduction of carbon emissions.

## Supporting information

S1 Data (XLSX)
